# Electrochemical measurement of serotonin by Au-CNT electrodes fabricated on microporous cell culture membranes

**DOI:** 10.1038/s41378-020-00184-4

**Published:** 2020-09-07

**Authors:** Ashley A. Chapin, Pradeep R. Rajasekaran, David N. Quan, Liangbing Hu, Jens Herberholz, William E. Bentley, Reza Ghodssi

**Affiliations:** 1 Fischell Department of Bioengineering, College Park, MD 20742 USA; 2 Institute for Systems Research, College Park, MD 20740 USA; 3 Department of Materials Science and Engineering, College Park, MD 20740 USA; 4 Department of Psychology and Neuroscience and Cognitive Science Program, College Park, MD 20740 USA; 5 Institute for Bioscience and Biotechnology Research, Rockville, MD 20850 USA; 6 Robert E. Fischell Institute for Biomedical Devices, Rockville, MD 20850 USA; 7 Department of Electrical and Computer Engineering, College Park, MD 20742 USA

**Keywords:** Carbon nanotubes and fullerenes, Electrical and electronic engineering

## Abstract

Gut–brain axis (GBA) communication relies on serotonin (5-HT) signaling between the gut epithelium and the peripheral nervous system, where 5-HT release patterns from the basolateral (i.e., bottom) side of the epithelium activate nerve afferents. There have been few quantitative studies of this gut-neuron signaling due to a lack of real-time measurement tools that can access the basolateral gut epithelium. In vitro platforms allow quantitative studies of cultured gut tissue, but they mainly employ offline and endpoint assays that cannot resolve dynamic molecular-release patterns. Here, we present the modification of a microporous cell culture membrane with carbon nanotube-coated gold (Au-CNT) electrodes capable of continuous, label-free, and direct detection of 5-HT at physiological concentrations. Electrochemical characterization of single-walled carbon nanotube (SWCNT)-coated Au electrodes shows increased electroactive surface area, 5-HT specificity, sensitivity, and saturation time, which are correlated with the CNT film drop-cast volume. Two microliters of CNT films, with a 10-min saturation time, 0.6 μA/μM 5-HT sensitivity, and reliable detection within a linear range of 500 nM–10 μM 5-HT, can be targeted for high-concentration, high-time-resolution 5-HT monitoring. CNT films (12.5 μL) with a 2-h saturation time, 4.5 μA/μM 5-HT sensitivity, and quantitative detection in the linear range of 100 nM–1 μM can target low concentrations with low time resolution. These electrodes achieved continuous detection of dynamic diffusion across the porous membrane, mimicking basolateral 5-HT release from cells, and detection of cell-released 5-HT from separately cultured RIN14B cell supernatant. Electrode-integrated cell culture systems such as this can improve in vitro molecular detection mechanisms and aid in quantitative GBA signaling studies.

## Sensors: Serotonin detection in cell culture

Researchers in the United States have developed a tool to rapidly and reliably detect serotonin at physiological concentrations. Serotonin is an important element in communication along the gut-brain axis, but measuring its concentration in the gut epithelium or derived cell cultures has proven challenging. A team at the University of Maryland, led by Reza Ghodssi, designed a system using carbon nanotubes deposited on gold electrodes to detect serotonin. The saturation time and detection range depended on the thickness of the nanotube film. By tuning the thickness, it was possible to create devices which could detect low concentrations with low time resolution or higher concentrations with a quicker saturation time. The new in vitro detection system can be used to investigate how serotonin is released by tissues along the gut-brain axis in healthy and diseased conditions.

## Introduction

Serotonin (5-hydroxytryptamine, 5-HT) is a neurotransmitter involved in neuronal synaptic signaling in the brain and peripheral nervous systems. The vast majority of 5-HT produced in the body is secreted within the gastrointestinal (GI) tract^1,2^, where it acts as a hormone and modulator of the enteric nervous system (ENS)^3,4^. Recent research and clinical evidence suggest that complex nervous communication occurs between the gut and the brain via the ENS and vagus nerve, termed the gut–brain axis (GBA)^5,6^. It is proposed that this communication is mediated by enterochromaffin cells (ECCs), which act as chemosensor cells within the gut epithelium by sensing luminal chemicals (e.g., allyl isothiocyanate—AITC^7^, short-chain fatty acids—SCFAs^8^) and secreting 5-HT from their basolateral side via secretory granule exocytosis^1,8–10^, thereby stimulating ENS and vagal afferent nerves at various 5-HT receptors^11,12^. Because of the inaccessibility of this tissue and the transient nature of molecular signaling, GBA serotonergic pathways are poorly understood^3,13^. While gut-on-a-chip systems have succeeded in modeling GI physiology in vitro^14^, integrating interfacial sensors for in situ detection of 5-HT proximal to gut tissue would facilitate monitoring of dynamic 5-HT release profiles in response to specific luminal stimuli.

5-HT detection and quantification are currently performed by a range of techniques, mainly benchtop assays (e.g., enzyme-linked immunosorbent assay [ELISA]), assays requiring sophisticated machinery (e.g., HPLC, mass spectrometry)^15,16^, and, more recently, electrochemical methods, which have been explored for in situ 5-HT monitoring. Electrochemical techniques present advantages over other gold-standard methods, owing to the use of simple electrodes for robust, quantitative molecular analysis. Electrodes are miniaturizable for use in a variety of settings, are able to be easily integrated with computational systems, and provide direct and label-free detection of specific redox molecules, depending on electrode modification. Existing electrochemical 5-HT sensors often use solid-state carbon materials such as glassy carbon or boron-doped diamond to achieve very-high-sensitivity detection in biological fluids^17–21^. Carbon microelectrodes have been used extensively for 5-HT detection in vivo in the brains of anesthetized animals^22,23^ and from ex vivo sections of the gut^24^. These applications are important for measuring real-time 5-HT release events from living tissue in response to various stimuli. However, in vivo electrode placement is very invasive, and measurements can be easily complicated by the complexity of the tissue environment. Importantly, in situ detection and other sampling methods^25^ have focused on 5-HT secreted into the gut lumen, but have not achieved quantification of 5-HT from the basolateral mucosa, where 5-HT stimulates enteric nerves and activates GBA pathways. In vitro platforms, particularly those that are based on redox information processing^26–28^, may provide better access than in vivo platforms to relevant molecules from cultured gut epithelial cells grown and tested in highly controlled environments^14,29–31^. Our in vitro platform, with electrochemical sensors embedded directly on the cell substrate, enables noninvasive and quantitative detection of 5-HT released from the basolateral side of a cultured gut epithelium or a secondary cell culture model thereof.

Cell-interfacial biosensor development requires biocompatible fabrication methods, using materials and architectures that maintain the integrity of biological systems to noninvasively obtain molecular information. However, fabrication of solid-state carbon electrodes relies on high temperature (800–1000 °C) and chemical processes^17,32^ that are not compatible with fabrication on delicate polymeric substrates, such as porous polyester track-etched (PETE) membranes. Facile drop-cast deposition methods use low temperatures and have minimal chemical and physical impact and thus are becoming more popular for integrating electrochemical sensing onto a variety of sensitive substrates^33,34^. Carbon nanotubes (CNTs) are widely used to modify electrochemical electrodes due to their high mechanical strength, excellent chemical stability, good biocompatibility, good electrical conductivity, and ability to serve as efficient signal transducers for redox molecule detection^35,36^. Their nanoscale structure allows CNT films to increase the electroactive surface area of electrodes, thereby increasing redox sensitivity. Graphitic structures, such as CNTs, are thought to adsorb aromatic molecules such as 5-HT via combinations of van der Waals forces and π–π stacking^35,37^. For these reasons, CNTs have been commonly used as electrochemical electrode modifiers to specifically detect 5-HT with high sensitivity and specificity^18,38^.

**Fig. 1:**
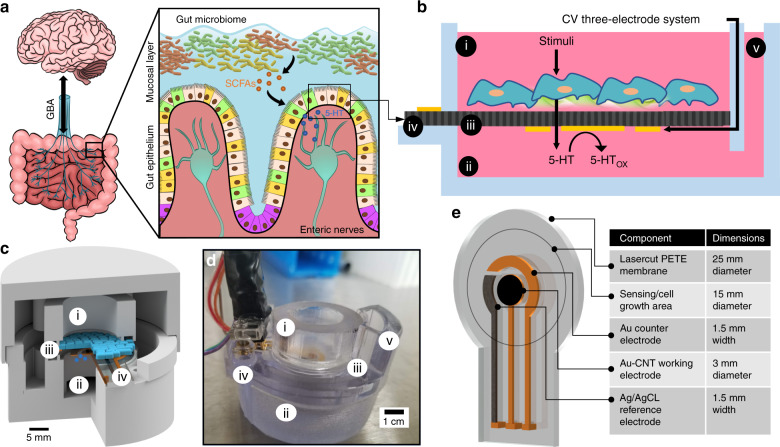
Diagram of electrochemical electrode-integrated in vitro platform and its potential utility to study the GBA. **a** Diagram of GBA physiology, depicting communication between the ENS and the brain (left). Diagram of luminal stimulation of enterochromaffin cells in the gut epithelium and subsequent basolateral 5-HT secretion to activate enteric nerves (right). **b** Conceptual diagram of how 5-HT would be detected from cultured gut cells in our platform. The cross-section is shown of our 3D-printed transwell system containing the (i) top chamber, (ii) bottom chamber, (iii) porous PETE membrane with integrated electrodes, (iv) external contacts, and (v) fluid inlet channel. The cyclic voltammetry (CV) electrodes are shown on the bottom side of the porous membrane to separate the cells from electronics via the insulating porous membrane. **c** Conceptual CAD diagram showing the 3D arrangement of the chambers sandwiching the electrode-integrated membrane. Cells grown on the membrane and basolaterally secreted 5-HT are shown in blue. **d** Image of a 3D-printed platform with pogo-pin connections at the contact pads used for testing in this paper. **e** CAD diagram of porous membrane fabricated with the CV three-electrode system. The sensing area is denoted with a circle, showing the hydrated part of the membrane that will be exposed to chemicals and eventually host a stable cell culture

In this work, we characterize CNT-modified Au electrodes on a PETE transwell membrane (Fig. 1) for dynamic, quantitative 5-HT detection in cell media. This membrane is used in transwell cell cultures and exhibits high porosity (16% by surface area) and a 1-µm pore size, which allows efficient molecular transport across the membrane while restricting mammalian cell transport or migration. Electrodes were fabricated on this membrane via metal (Ti/Au/Ag) electron beam evaporation and then subsequent drop-casting of CNT films, where the film thickness correlated with both the sensitivity (0.6 µA/µM and 4.5 µA/µM for 2 µL and 12.5 µL CNT films, respectively) and the saturation time (10 min and 2 h, respectively). We achieved 5-HT detection at concentrations relevant to those secreted from ex vivo sections of the gut mucosa^24^ in the 100 nM–10 μM range across the two electrodes tested. Dynamic detection of 5-HT diffusion through the porous membrane was demonstrated, mimicking the burst mode of molecular release that is observed from cells via secretory granule exocytosis^39^. The facile, biocompatible assembly process, porous nature of our electrodes, and subsequent electrochemical monitoring capabilities of this platform introduce a new molecular detection modality for integration with in vitro systems. GOC, organ-on-chip, and organoid systems can potentially benefit from this direct redox-enabled electrochemical-biological interface, including the study of dynamic 5-HT release profiles from model tissues within the GBA and how these profiles are stimulated by healthy and disease-state GI conditions.

## Results

### Characterization of carbon nanotube (CNT) coatings

**Fig. 2:**
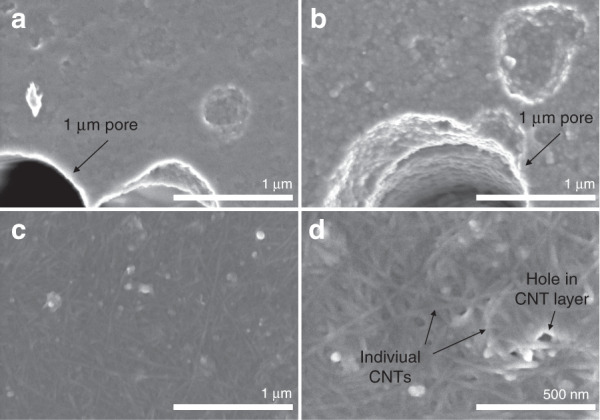
SEM images of CNT-film coatings on select PETE membranes. **a** Bare PETE membrane. **b** PETE membrane-coated with Au. **c**, **d** Au-PETE coated with 12.5 µL of CNT at two magnifications. Membrane pores, CNTs, and CNT-film defects are indicated with arrows

Before full assembly and testing of the 3D-printed platform, SEM analysis was performed to evaluate the nanostructure of the CNT film on a Au-coated porous PETE membrane (Fig. 2). Both bare PETE and Au-PETE clearly showed 1-µm pores in the membrane, demonstrating that the thin 100-nm Au coating does not block the pores (Fig. 2a, b). The Au coating added a slight granular texture to the flat polymer surface (Fig. 2b). Drop-casting a CNT film on the Au-coated PETE (12.5 µL of the described single-walled CNT [SWCNT] solution) (Fig. 2c, d) produced a thick, dense mesh structure with nanoporosity that would be expected to provide a significantly higher surface area than bare Au. The width of the individual SWCNTs appears to be ~20–50 nm, with lengths in the micron range, demonstrating very-high-aspect-ratio nanostructures that contribute to the electroactive surface. Defects in the layer, including impurities and holes, can potentially act as added binding sites for 5-HT or other molecules^40^. The CNT-film morphology seen here is expected to be highly electroactive. The electroactivity of these CNT films was assessed across a range of drop-cast volumes on both microdisk and membrane Au electrode substrates (Supplementary Fig. S3). From this, we continued testing 2 µL CNT coatings on microdisk electrodes, and membrane electrodes were tested with the thin 2 µL film and the thick 12.5 µL film (Supplementary Fig. S4).

**Fig. 3:**
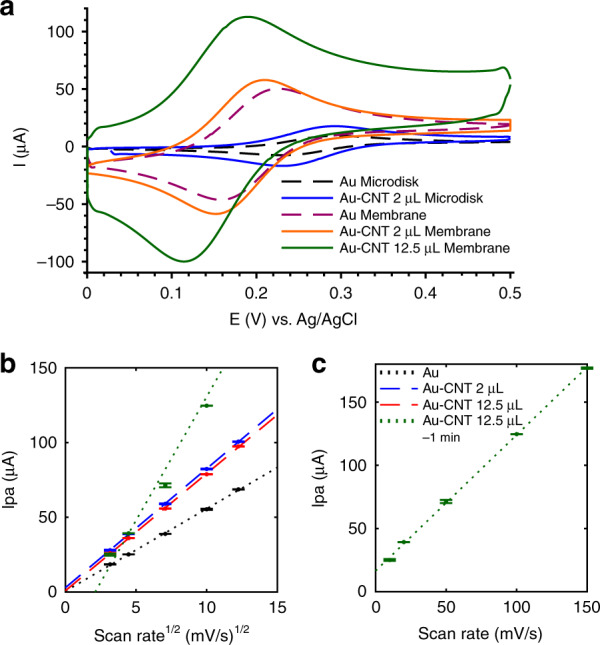
Characterization of electrode CNT modifications by CV detection of 2 mM ferrocene dimethanol (FDM). **a** Representative cyclic voltammograms of bare Au and CNT-coated microdisk and membrane electrodes. **b**, **c** Membrane electrodes were tested for scan-rate dependence at the following levels of CNT modification: bare, 2 µL of CNT coating, and 12.5 µL of CNT coating, which exhibited linear behavior of Ipa vs. $\sqrt {\it{\upnu }}$ (**b**). The Au-CNT (12.5 µL) membrane electrode showed a time-dependent reaction to 2 mM FDM, so a 1-min accumulation time was allowed between CV cycles. The obtained Ipa data are plotted against $\sqrt {\it{\upnu }}$ in (**b**) to compare with other coatings and plotted against ${{\upnu }}$ in (**c**) to obtain a better linear fit. The scan rates were 10, 20, 50, 100, and 150 mV/s (*n* = 3 cycles per electrode). **b**, **c** share the same legend

Figure 3a shows a comparison of each electrode type, with and without CNT coatings, for their cyclic voltammetric (CV) current response to 2 mM ferrocene dimethanol (FDM). The anodic and cathodic peak voltages were consistent between the Au and Au-CNT microdisk electrodes (Epa ~ 0.29 V, Epc ~0.23 V), but shifted left when measured at the Au membrane electrodes (Epa ~0.22 V, Epc ~0.16 V) and continued to shift with increased CNT coating on the membrane (Epa values: 0.2 V and 0.19 V for 2 µL and 12.5 µL, respectively). Nernstian behavior is indicated by near-ideal anodic and cathodic peak separation (Epa-Epc ~0.060–0.068 V, where the ideal value is 0.059 V for a one-electron-transfer reaction) and peak current ratio (Ipa/Ipc ~0.81–1.16, where the ideal ratio is ~1 for a perfectly reversible reaction^41^). The current peaks (Ipa and Ipc) increased due to the CNT coating in both microdisk and membrane electrodes, where 12.5 µL of CNT produced a higher current than 2 µL of CNT on membrane electrodes.

The scan-rate dependence was assessed across uncoated and CNT-coated Au membrane electrodes (Fig. 3b, c) to characterize the mode of FDM detection and the influence of the CNT-coating thickness. The Randles–Ševčik Eq. (1) describes the linear correlation between the peak current (Ipa) and the square root of the scan rate ($\sqrt \nu$) in diffusion-limited reversible systems^41^.

1 $${\mathrm{Ipa}} = 0.446\,nFAC\sqrt {\frac{{nFD\nu }}{{RT}}},$$

where *n* is the number of electrons transferred, *F* is Faraday’s constant, *A* is the electrode surface area, *C* is the solute concentration, *D* is solute diffusion constant, ${\it{\upnu }}$ is the scan rate, *R* is the ideal gas constant, and *T* is the temperature. This linear correlation was seen in Fig. 3b for each electrode, confirming that the ferrocene redox reaction was diffusion limited and reversible. The current response over the scan rate takes the form of the following linear equations: Au membrane: ${\mathrm{Ipa}} = 5.52\sqrt \nu + 0.52$ (*R*^2^ = 0.9994), Au-CNT (2 µL) membrane: ${\mathrm{Ipa}} = 7.96\sqrt \nu + 2.89$ (*R*^2^ = 0.9999), Au–CNT (12.5 µL) membrane: ${\mathrm{Ipa}} = 7.84\sqrt \nu + 0.85$ (*R*^2^ = 0.9998).

As described by the Randles–Ševčik Eq. (1), the slope of the line is proportional to the effective surface area (ESA) of the electrode^42,43^. It can be seen that the presence of the CNT coating on the Au membrane electrode increased this slope, thereby showing an increased ESA. However, no difference can be seen between the 2 µL and 12.5 µL coatings, except when a 1-min accumulation time was applied between each CV cycle. By varying the accumulation time between CV cycles during 2 mM FDM detection (Supplementary Fig. S5), it was seen that ferrocene molecules needed ≥15 s to fully diffuse into the 12.5 µL CNT film to saturate the signal. Indeed, applying a 1-min accumulation time during the scan-rate sweep resulted in a linear relation with an increased slope (${\mathrm{Ipa}} = 16.5\sqrt {\upnu} - 31.8$, *R*^2^ = 0.9816), demonstrating a higher ESA than that of the 2 µL film (Fig. 3b). However, these data better fit an adsorption-controlled process where Ipa varies linearly with scan rate (${\mathrm{Ipa}} = 1.075{\upnu} + 16.5$, *R*^2^ = 0.9994). The transition from diffusion-controlled to adsorption-controlled behavior indicated the increased capacity for molecular binding and retention at the 12.5 µL CNT electrode, as has been shown to occur for other electroactive films^44^.

### 5-HT detection at CNT-modified membrane electrodes

**Fig. 4:**
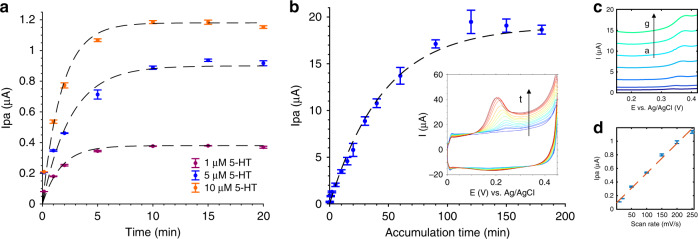
Accumulation-time- and scan-rate-dependent detection of 5-HT at Au-CNT membrane electrodes. **a**, **b** Accumulation-time-dependence of 5-HT detection for (**a**) 2 µL and (**b**) 12.5 µL of CNT coatings of Au membrane electrodes. 5-HT concentrations measured in (**a**) include 1, 5, and 10 µM (*n* = 3 cycles per electrode), while (**b**) corresponds to 5 µM (*n* = 2 cycles per two electrodes). Inset: CV curves measured over increasing accumulation times, indicated by the arrow. **c** Scan-rate dependence of the Au-CNT (2 µL) membrane electrode measuring 500 nM 5-HT using a 20-min accumulation time between CV cycles. The scan rate was 10, 20, 50, 100, 150, 200, and 250 mV/s (**a**–**g**). **d** Ipa data are plotted against the scan rate and fit with a line (*n* = 2 cycles per measurement)

The CNT-coated membrane electrodes were then characterized for their sensitivity to 5-HT, although it was determined that the adsorption-limited nature of 5-HT electrochemical detection^20,21^ creates a time dependency in electrode sensitivity. Figure 4 demonstrates the time-dependent nature of Au-CNT membrane electrode detection of static 5-HT solutions at constant concentrations, without the input of stirring or heating. Over increasing accumulation times between CV cycles, Ipa increased until saturation was achieved, indicating that 5-HT molecules adsorb on the electrode over time. As shown in Fig. 4a, b, respectively, membrane electrodes coated with 2 µL CNTs saturated after ~10 min, regardless of the 5-HT concentration, while 12.5 µL CNT-coated electrodes saturated after ~2 h. This trend can be modeled as a first-order system response over accumulation time *t*_acc_:

2 $$I_{pa}\left( {t_{{\mathrm{acc}}}} \right) = A\left( {1 - e^{ - \frac{{t_{{\mathrm{acc}}}}}{\tau }}} \right),$$

where *A* is the maximum *I*_*pa*_ signal achieved at steady state and *τ* is the time constant of the curve, equal to the time required to reach 63.2% of the steady-state value^45^. Fitting this equation to the data in Fig. 4a, b allows estimation of *A* and *τ* for each electrode: Au-CNT (2 µL) membrane, [5-HT] = 1, 5, 10 µM: *A* = 0.47, 1.06, 1.44 µA and *τ* = 2.76, 2.38, 1.58 (*R*^2^ = 0.834, 0.969, 0.965); Au–CNT (12.5 µL) membrane, [5-HT] = 5 µM: *A* = 20.3 µA and *τ* = 52.58 (*R*^2^ = 0.990). These signal saturation rate constants are approximately similar for the Au-CNT 2 µL electrodes, although an increase in 5-HT concentration correlates with higher *A* values and smaller *τ* values, resulting in saturation at higher signals over ~10 min. These rate constants are increased by more than one order of magnitude for the Au-CNT 12.5 µL electrode, indicating saturation at a significantly higher signal over ~2 h. Considering the relation of this process to an RC circuit, *τ* can be approximated to equal the product of the resistance and capacitance^45^. It stands to reason that the increase in *τ* associated with the Au-CNT 12.5 µL electrode is due to an increased capacitance for 5-HT binding within the thicker CNT film. Further, if we compare the linear range of the two Au-CNT electrodes, the 12.5 µL CNT electrode has a linear range of ~0–30 min accumulation time, a 15 times increase over the ~0–2-min linear range of the 2 µL CNT electrode. However, when comparing 5 µM 5-HT detection at both electrodes, the slopes of their linear ranges are found to be approximately the same (0.2 µA/min and 0.3 µA/min for the 2 µL and 12.5 µL CNT-coated electrodes, respectively). The slopes of the signal over time may be the same for these films due to a constant binding rate of 5-HT on the SWCNTs, which would control the kinetics of accumulation. However, a higher overall signal can be achieved at the thicker film because there are more binding sites available for 5-HT.

To support the theory introduced in Fig. 4a, b that 5-HT detection is adsorption controlled, the scan-rate dependence was assessed by measurement of 500 nM 5-HT at the Au-CNT (2 µL) membrane electrode (Fig. 4c, d). In this case, Ipa varied linearly with the scan rate according to the following equation: ${\mathrm{Ipa}} = 0.004\nu + 0.092$ (*R*^2^ = 0.9928). This follows the standard Eq. (3), which describes that adsorption-controlled reactions are linear with respect to Ipa and *v*:

3 $${\mathrm{Ipa}} = \frac{{n^2F^2}}{{4RT}}\,\nu \,\Gamma _{eq}.$$

In this scheme, Ipa is related to both the scan rate and the equilibrium surface coverage $\Gamma _{eq}$, which is a function of the 5-HT concentration and electrode ESA.

**Fig. 5:**
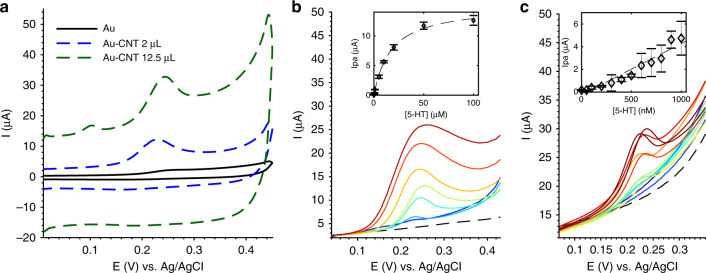
5-HT sensitivity demonstrated at Au and Au-CNT membrane electrodes. **a** Representative CVs of 5 µM 5-HT measured at each electrode. **b** Au-CNT (2 µL) membrane electrode sensitivity to a range of 5-HT: 500 nM–100 µM using an accumulation time of 10 min. The inset shows a Langmuir curve fit (*n* = 2 cycles per two electrodes). **c** Au-CNT (12.5 µL) membrane electrode sensitivity to a range of 5-HT: 10 nM–1 µM using an accumulation time of 2 h. A line fit was applied to the data (*n* = 2 cycles per two electrodes)

Concentration-dependent detection of 5-HT was then compared using both Au-CNT membrane electrodes, as shown in Fig. 5. Compared with Au membrane electrodes, which showed a broad CV peak and very low current response to 5-HT, CNT coatings increased peak sharpness and height while maintaining an Epa of ~0.22–0.23 V (Fig. 5a). The sensitivity of each Au-CNT membrane electrode was evaluated in Fig. 5b, c, in which the 2 µL CNT electrode was used to detect higher 5-HT concentrations (500 nM–100 µM) with a shorter accumulation time (10 min), and the 12.5 µL CNT electrode was used to detect lower 5-HT concentrations (10 nM–1 µM) with a longer accumulation time (2 h). Figure 5b shows that the 2 µL-coated electrode attained a linear range of 0.5–10 µM, where the slope of the linear region denotes sensitivity: 0.6 µA/µM (*R*^2^ = 0.9976). The resolution of detection can be calculated by 3*σ (σ: standard deviation of the lowest concentration): 3*0.0068 µA = 0.024 µA. From this, the limit of detection (LOD) can be calculated as resolution/sensitivity = 30 nM. The limit of quantitation (LOQ) can be calculated as 10*σ/sensitivity = 100 nM. In comparison, Fig. 5c shows that the 12.5 µL-coated electrode attained a linear range of 0.1–1 µM and a sensitivity of 4.5 µA/µM (R^2^ = 0.9993). The resolution calculated as 3*σ is 3*0.046 µA = 0.138 µA, resulting in an LOD of 30 nM and LOQ of 100 nM. Per these calculations, both electrodes achieve the same LOD and LOQ; however, in practice, the Au-CNT 2 µL electrode does not produce noticeable peaks below ~500 nM 5-HT. The sensitivity of the Au-CNT 12.5 µL electrode is 7.5× that of the Au-CNT 2 µL electrode when using a 2-h accumulation time (12× longer than 10 min for the 2 µL electrode). This demonstrates that thicker CNT films can be used to measure low 5-HT concentrations with low time resolution, while thinner CNT films are useful for high-concentration and high-time-resolution applications. However, the low-concentration measurements in Fig. 5c vary more in peak height and voltage than the high-concentration measurements, as indicated by large error bars, and so it may be difficult to distinguish very low concentrations.

### Dynamic 5-HT monitoring

**Fig. 6:**
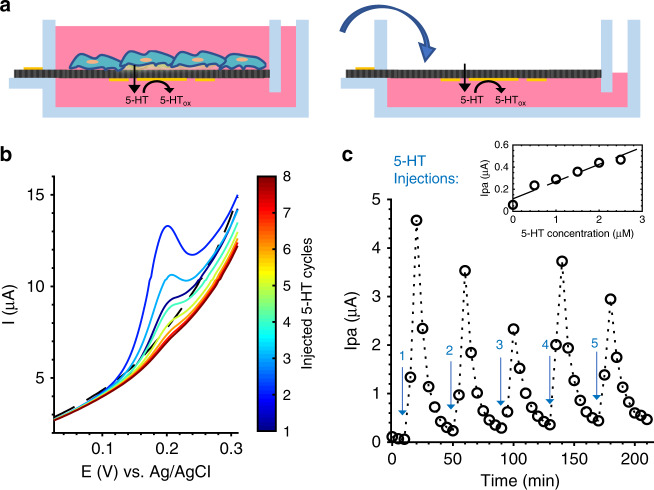
5-HT injection experiment—dynamic CV detection of 5-HT bursts and diffusion over time through a porous membrane. **a** Schematic illustrating the mode of 5-HT release from cells (left) and our injection experiment, which simulates this mode of 5-HT release by repeated injections (right), which was monitored at an Au-CNT (2 µL) membrane electrode. **b** Representative CV curves over the course of one injection event, centered on the peaks. DMEM blank—black, dashed line. 5-HT injection—color denotes the cycle (1–8) with 5-min accumulation time between each cycle. **c** Full timeline of Ipa values generated by five injections of 5-HT, 40min apart, denoted by arrows. Inset: linear trend between the final Ipa value per injection and expected final 5-HT concentration: 0, 0.5, 1, 1.5, 2, 2.5 µM

5-HT is released from ECCs via vesicle exocytosis, resulting in a burst of 5-HT near the site of release and then diffusion to surrounding areas^24^. To demonstrate the ability of our device to monitor this type of dynamic release, we simulated burst release by injecting known concentrations of 5-HT just above the porous membrane and monitoring the change in 5-HT concentration with CV electrodes over the course of diffusion through the membrane and into the bulk volume (Fig. 6). A schematic of this cell-release simulation via 5-HT injections is illustrated in Fig. 6a. Five 17.5 µL injections of 100 µM 5-HT were monitored in this process with 5-min accumulation time per measurement. Each injection, when fully diffused into 3.5 mL Dulbecco’s modified Eagle medium (DMEM) bulk volume, was expected to reach a final 5-HT concentration of 0.5 µM, wherein each subsequent injection would increase the final bulk concentration by 0.5 µM (ranging from 0.5 to 2.5 µM over the course of the experiment). A 2 µL CNT-coated electrode was used for this experiment to obtain a fast time resolution. The timeline of Ipa values detected from these injections shows spikes at 10 min after the injection, followed by an exponential decrease in Ipa until the next injection event, when 5-HT detection spikes again. Previous work by our group^46^ showed that monitoring FDM diffusion through the membrane yielded a peak in Ipa at 1 min after injection, indicating that small-molecule transport across the membrane occurs within this 1-min timescale. 5-HT diffusion should occur at a similar or faster timescale, as it is a smaller molecule (5-HT: 176 Da, FDM: 246 Da); however, it resulted in a peak in Ipa at 10 min after injection. As demonstrated in Fig. 4a, this may be due to the time dependency of 5-HT adsorption and reaction at the Au-CNT electrode, where it was shown that molecular saturation occurs at ~10 min in a constant-concentration solution. The exponential decrease seen after the Ipa peak follows the expected decrease in concentration due to 5-HT diffusion away from the electrode. A linear trend can be observed by plotting the final Ipa values per injection against the expected final concentration (Fig. 6c, inset) with a slope of 0.156 µA/µM. There was significant variation in the peak Ipa values for each injection, despite using a constant concentration of 5-HT. However, since detection of 5-HT at constant concentrations has been demonstrated to be highly repeatable (denoted by *R*^2^ values in Fig. 4, 5), this may be due to experimental error when placing the pipet at the membrane surface for each injection or because our measurement frequency is much slower than the diffusion dynamics. The consistent shape of each injection curve provides confidence that dynamic monitoring of 5-HT burst release is feasible within this system, owing to the close proximity of electrodes to the cell culture membrane where release events would occur.

### RIN14B enterochromaffin cell-released 5-HT

**Fig. 7:**
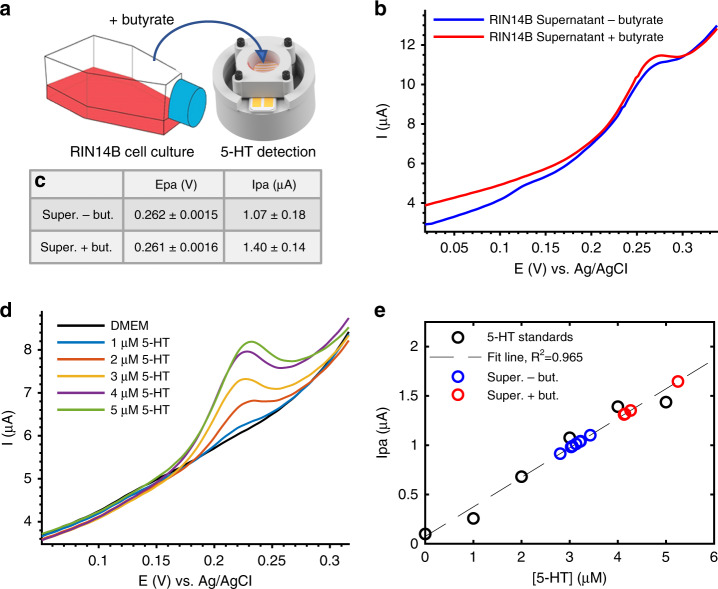
Detection of 5-HT from RIN14B cells cultured in a T75 flask. **a** Illustration of cell supernatant transferred from the T75 flask cell culture to the Au-CNT (2 µL) electrode-integrated membrane platform for CV measurement. **b**, **c** CV measurement of cell supernatant before (−) and after (+) stimulation with butyrate. **b** Representative CV curves. **c** Epa and Ipa values obtained from all curves in (**b**) (*n* = 5 cycles per measurement). **d** Electrode calibration with 5-HT standards in DMEM. **e** Linear fit of 5-HT standards (*n* = 3 cycles per measurement). Ipa values measured from the supernatant before and after butyrate stimulation are fit on the line to approximate 5-HT concentration in each sample: ~3.1 µM before (−) butyrate, ~4.1 µM after (+) butyrate (excluding outlier). A 5-min accumulation time was used for all CV measurements

Detection of 5-HT secreted from a standard T75 flask culture of an immortalized enterochromaffin-model cell line (TRPA1-expressing RIN14B cells) was also achieved, demonstrating that cell-secreted 5-HT is detectable within the linear range of our electrode. Figure 7a depicts the detection of 5-HT in the supernatant of RIN14B cells cultured in a separate T75 flask, as measured at 2 µL Au-CNT membrane electrodes. Cells were stimulated to release 5-HT by 1 h incubation with 100 µM sodium butyrate, a short-chain fatty acid produced by commensal gut bacteria that is known to stimulate enteric 5-HT production in vivo and in RIN14B cells^2^. Figure 7b, c shows that the CV signal increased by ~0.33 µA at Epa ~0.26 V, indicating that the 5-HT concentration detected in the supernatant increased after butyrate stimulation. Background 5-HT is detected in the cell supernatant before stimulation, potentially due to basal 5-HT release from cells or due to the presence of 5-HT in fetal bovine serum (FBS), which varies from batch to batch. A calibration curve was performed using the same electrode with 5-HT standards (1–5 µM 5-HT in DMEM) as an internal control to estimate the concentration of cell-released 5-HT (Fig. 7d). When compared with this calibration curve, the approximate concentrations of 5-HT detected from the supernatant before and after stimulation were ~3.1 µM and ~4.1 µM, respectively, suggesting that ~1 µM 5-HT was released from the cells. This cell culture contained 25 × 10^6^ cells in a 5 mL volume, corresponding to 0.2 nmol 5-HT released/10^6^ cells. This result demonstrates that RIN14B cells can be stimulated to release 5-HT using a known microbial metabolite and that this cell-released 5-HT can be detected by our Au-CNT membrane electrode in the cell supernatant. Equally importantly, no other redox active molecules were found in this cell supernatant within the ranges of the applied CV scan. This greatly enhances our ability to attribute biological function to 5-HT concentration. In future experiments, it could be tested whether other biological redox molecules (e.g., ascorbic acid, uric acid) interfere with 5-HT detection by producing overlapping CV peaks. The use of nanostructured electrode coatings has been shown to separate these peaks to distinguish between 5-HT and other contaminants. The CNT film used here may provide this capability^38^ or may be further optimized in combination with graphitic structures^19^, nanostructured platinum^47^, and ionic polymers such as Nafion^38^ and chitosan^48,49^.

**Fig. 8:**
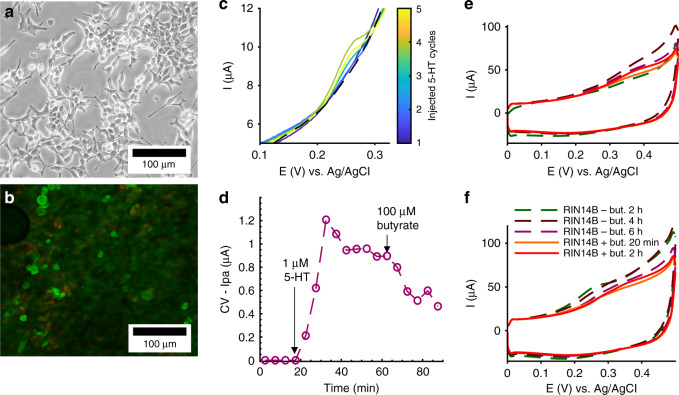
Poor membrane attachment of RIN14B cells is a limiting factor for 5-HT detection from cells cultured on our electrode-integrated membrane **a, b** Optical micrographs of RIN14B cells. **a** Bright-field image of RIN14B on a T75 polystyrene flask. **b** Confocal fluorescence microscopy of live/dead stained RIN14B grown on an electrode-integrated cell culture membrane-coated with collagen. Live cells: green (Syto9), dead cells: red (propidium iodide). The cell morphology and density were analyzed. **c**, **d** RIN14B cells cultured on a Au-CNT (2 µL) membrane electrode coated with collagen were monitored over the course of molecular treatment, where **c** shows CV curves and **d** shows Ipa values measured from those curves. **c** CV curves show baseline (black—dashed) and exogenously injected 5-HT (color bar). **d** Time course of Ipa values, where arrows denote time of injections: spiking with 1 µM 5-HT for calibration, and stimulation with 100 µM butyrate. A 5min accumulation time was used between CV cycles. **e**, **f** RIN14B cells cultured on Au-CNT (12.5 µL) membrane electrodes, **e** with collagen and **f** without collagen, were monitored with a 2 h accumulation time between CV cycles. Dashed lines denote baseline measurements taken at *t* = 2, 4, and 6h. Solid lines denote measurements taken at *t* = 20 min and 2 h after the addition of 100 µM butyrate

RIN14B cells were plated and cultured directly on the electrode-integrated membrane and characterized by optical microscopy and CV monitoring, as shown in Fig. 8. Neither the 3D-printed platform in which cells were plated nor the PETE membrane used here was optically transparent, so assessment of the cell culture was performed with endpoint live/dead fluorescent staining and confocal microscopy. Figure 8a, b shows representative images of RIN14B cells plated on polystyrene T75 flasks (Fig. 8a), imaged with bright-field microscopy, and RIN14B cells plated on an electrode-integrated PETE membrane (Fig. 8b), imaged with fluorescence microscopy. The cells plated on polystyrene have epithelial-like morphology with many long processes and are clearly well attached to the surface. Comparatively, the cells plated on the porous membrane are rounded and sparse and appear to lack strong attachment to the membrane, despite collagen treatment.

In Fig. 8c, d, an Au-CNT (2 µL) membrane electrode was used to monitor cells over the course of molecular treatment of the cell culture, including injections of 1 µM 5-HT to calibrate the electrode and spike the solution above the LOD of the electrode (denoted by the arrow). In Fig. 8d, compared with the cell-only baseline over *t* = 0–20 min, which showed no peaks, the 1 µM 5-HT spike at *t* = 20 min induced an increase in Ipa over *t* = 20–35 min that then gradually decreased until *t* = 60 min. The increased time to reach a peak in Ipa, compared with the no-cell measurements in Fig. 6c, can be attributed to many factors, including the cell barrier and the collagen barrier. A subsequent injection of 100 µM butyrate did not lead to an appreciable increase in Ipa, although the shape of the graph does not decrease exponentially as it does in Fig. 6c. This may indicate a small, immeasurable release of 5-HT. Similar results are seen in Fig. 8e, f, in which RIN14B cells were monitored on Au-CNT (12.5 µL) membrane electrodes that were either coated with collagen (Fig. 8f) or not (Fig. 8e). Cells were measured at 6, 4, and 2 h timepoints before treatment with butyrate to establish a baseline and then 20 min and 2 h after butyrate treatment to measure any stimulated 5-HT release. All CV curves before and after butyrate treatment showed no appreciable 5-HT secretion despite using 2-h accumulation times, which would maximize the signal and allow for a sub-100-nanomolar detection range. These results suggest that RIN14B cell attachment to the 1-µm pore PETE membrane was not strong enough to produce a sustainable, healthy cell culture, nor is it conducive to the cell pathways required for 5-HT secretion. However, these results indicate that healthy cell cultures are sustainable on polystyrene plastics, and the electrodes are still capable of 5-HT detection in the presence of collagen and cells.

## Discussion

### Enhanced detection capabilities at CNT-coated electrodes

In this work, two types of CNT films were deposited on porous transwell membranes and compared regarding their molecular detection capabilities. The thicker CNT coating (12.5 µL) showed a higher electroactive surface area as determined by the increased slope of Ipa vs. $\sqrt {\upnu}$, which increased the current response to FDM and 5-HT compared with that of the thinner coating (2 µL) and bare Au electrodes, but this effect was determined to be time dependent for the detection of both molecules. Longer diffusion times (~2 h) were required to allow full signal saturation of the thicker film, resulting in a lower LOD of 5-HT ~30 nM. In contrast, the thinner CNT film more quickly achieved full signal saturation, and it did not require added time for FDM diffusion into the film. 5-HT still required ~10 min for full signal saturation, perhaps due more to the adsorption-limited nature of the molecule than diffusion into a thick nanoporous mesh. The thin film was also able to detect a wide linear range of 5-HT concentrations that are relevant to physiological tissue, although in practice, the LOD was higher than that of the thicker film ~500 nM. These concentrations are well within the expected micromolar concentration range of 5-HT release detected from the apical side of ex vivo GI tissue^24^.

Both films demonstrated a similar time resolution of 5-HT detection, as denoted by a nearly identical slope of Ipa/accumulation time. However, the thick film showed a linear increase in signal over a wider range of accumulation times (up to 30 min), giving a greater ability to tune the sensitivity and sampling frequency for the specific need of molecular detection. The thin film is more useful for the dynamic detection of 5-HT injections. Injections at this electrode took 10 min to reach a peak value, suggesting that the saturation time constant (i.e., 63.2% of the time to reach saturation) might be a good measurement frequency for approximating concentrations in real time. Although a 10 min frequency (or even 63.2% of this) is too slow to detect the sub-second timescale of individual ECC 5-HT release events^39,50^, it would be able to monitor broad fluctuations in secreted 5-HT concentrations in response to varied cell treatments with higher frequency and less handling than offline methods such as ELISA or HPLC. Higher-frequency continuous monitoring can be achieved in future work by tuning the electrode coating to decrease the saturation time.

### 5-HT detection from RIN14B cells

RIN14B cells cultured in T75 flasks were stimulated with butyrate to produce measurable 5-HT. Approximately 1 µM 5-HT was produced over 1 h from a confluent cell layer of 25 × 10^6^ cells, corresponding to 0.2 nmol 5-HT released/10^6^ cells. The feasibility of 5-HT detection from RIN14B cells cultured directly in our in vitro platform can be determined by extrapolating this 5-HT release concentration. The surface area available for cell attachment on the porous membrane is 2 cm^2^ (as opposed to the 75 cm^2^ in T75 flasks), allowing for a maximum of 0.67 × 10^6^ cells at confluency, assuming ideal conditions. Given the 5-mL device volume, we can calculate the 5-HT released from a confluent cell layer to be ~24 nM, below the threshold of electrode detection. However, a main benefit of incorporating electrodes directly on the porous membrane in direct contact with cells is access to high local 5-HT release concentrations. If we consider detection of 5-HT released just from cells plated above the Au-CNT working electrode (0.023 × 10^6^ cells above the 3-mm-diameter CNT-modified WE) and assume 5-HT will be released into the 16.8-nL total pore volume within the WE, the maximum 5-HT concentration achieved at the electrode would be ~274 µM, which would be significantly above the detection threshold, demonstrating the feasibility of the cell-monitoring system. Currently, the limiting factor is the difficulty in culturing RIN14B cells on a 1-µm PETE membrane, as described further in the supplementary information. In essence, RIN14B cell attachment suffers from the high porosity of membranes such as this PETE membrane. Attachment can be enhanced by modifying the membrane pore size and distribution as well as optimizing surface properties by plasma activation or coating with other extracellular matrix proteins such as laminin or collagen IV.

Given the electrochemical 5-HT detection capabilities of the electrodes assessed here, this system can perform continuous, concentration-specific 5-HT detection directly on a porous polymeric membrane. Future work to stabilize the culture of RIN14B model ECCs on transwell substrates will be beneficial by providing high spatial proximity for superior access to molecular information that can be easily lost in complex tissues. Customizable molecular measurement capabilities, such as controlling the concentration range and time resolution, are also beneficial to developing protocols for studying unknown cellular signaling interactions, such as basolateral 5-HT signaling underlying GBA communication.

## Materials and methods

### Fabrication of electrodes on a porous cell culture membrane

A full description of the fabrication of the 3D-printed platform and electrode fabrication on a porous membrane is detailed in Rajasekaran et al.^46^. Briefly, CV electrodes were fabricated on a porous polyethylene track-etched (PETE) membrane (1 µm pore diameter, 16% porosity, 11 µm thickness) (Sterlitech, Kent, WA, USA). E-beam evaporation of 20-nm Ti, 100-nm Au, and 500-nm Ag metals was utilized for electrode deposition with a paper shadow mask for patterning, and Ag reference electrodes were chemically converted to Ag/AgCl for CV measurements (Fig. 1e). The CV electrodes were fabricated with an outer diameter of 8.5 mm, counter and reference electrode width of 1.5 mm, and working electrode diameter of 3 mm. Each electrode connected to a rectangular contact pad, allowing all electrodes to be contacted by pressing pogo pins (Digikey, Thief River Falls, MN, USA) onto the top-side contact pads. After electrode patterning, the membrane was sealed inside the 3D-printed platform by curing polydimethylsiloxane (PDMS) around the outside 5 mm section of the membrane (Fig. 1c, d). The internal 15 mm diameter was exposed to fluidic infill of the top and bottom chambers, which is denoted as the sensing area in Fig. 1e. PDMS was cured at 60 °C for 3 h.

### CNT-Au electrode modification for 5-HT CV detection

The 3-mm-diameter circular part of the CV working electrode (WE) was modified with CNTs to enhance 5-HT binding. A challenge of CNT electrode modification is the ability to uniformly disperse CNTs in solution and then cast a stable CNT film on the electrode surface. This is generally approached by dispersing CNTs in a hydrogel or polymer matrix and entrapping them on the surface;^38^ however, this lowers the CNT density and often requires the addition of further electroactive and catalytic materials to maintain high sensitivity. Here, we achieve a very uniform CNT dispersion in a 1:1 mixture of N-methyl-2-pyrrolidone (Fisher Scientific), an organic solvent, and ethanol (NMP•EtOH), followed by ultrasonication which produces a highly concentrated CNT film when drop-cast.

Purified SWCNTs were purchased from Carbon Solutions Inc. (no. P3-SWNT) (Riverside, CA, USA), which are highly functionalized with 1–3 atomic % carboxylic acid groups. A 1 mg/mL SWCNT solution in 1:1 NMP•EtOH was prepared by Dr. Liangbing Hu’s laboratory. Different volumes of the solution were drop-cast onto the Au working electrode, and the solvent was evaporated using a heat gun set to 454 °C at low speed. The PETE membrane can melt at a sustained temperature exceeding 140 °C, but heat applied by the heat gun was not held for long enough (~30 s) to damage the membrane. A Hitachi SU-70 SEM was used to observe the presence of the CNT modification on an Au-coated PETE membrane (Fig. 2).

### Cyclic voltammetry electrochemical measurement procedures

Standard Au microdisk electrodes were used to first characterize CNT electrode modification. Au electrodes 2 mm in diameter were purchased from CH Instruments (Austin, TX, USA), along with alumina polishing materials. Au electrodes were first polished with alumina powders of 3-µm, 1-µm, and 0.05-µm grain sizes. The electrodes were then ultrasonicated to remove excess powder. The electrodes were either left bare for testing or coated with the 1 mg/mL SWCNT solution, as described above. These electrodes were used as working electrodes and were tested alongside a Pt coil counter electrode and Ag/AgCl reference electrode in saturated KCl, all of which were purchased from CH Instruments.

Microdisk and membrane electrodes underwent cyclic voltammetry (CV) electrochemical measurements using a VSP 300 potentiostat, and data were recorded using EC-Lab software from Bio-Logic Science Instruments (Seyssinet-Pariset, France). CV was performed at a scan rate of 50 mV/s, unless otherwise stated, scanning in the range of −0.05–0.45 V for 1,1’-ferrocene dimethanol (FDM, Sigma-Aldrich) measurements and scanning over 0–0.45 V for 5-HT (Alfa Aesar, Ward Hill, MA, USA) measurements. The CV current was graphed in response to the potential applied at the working electrode, in reference to the potential of the reference electrode, labeled ‘E(V) vs. Ag/AgCl’. Both FDM and 5-HT measurements were performed in Dulbecco’s modified Eagle’s medium (DMEM) (Sigma-Aldrich, St. Louis, MI, USA), which was the medium used to culture cells, as described below. Membranes were sealed inside the 3D-printed platform with CV electrodes facing down when testing dynamic 5-HT monitoring or when monitoring release from cultured cells to keep the cells insulated from the voltages and currents produced by CV. In this case, contact was made between pogo pins and Au pads on the top side of the membrane, which were electrically connected to the contact pads on the bottom by sticking a needle through the two pads and flowing Ag ink into the hole. For all other experiments, the membrane was oriented with electrodes facing up for ease of access and contact.

5-HT fouling is a well-known phenomenon in that oxidation products readily adsorb and polymerize on electrodes, blocking available binding sites for new 5-HT binding and detection^20^. For this reason, repeated polishing was necessary for Au microdisk electrodes. However, this was not possible for Au-CNT electrodes or membrane electrodes, and therefore, these electrodes were replaced when fouled. We plan to characterize the full impact of fouling on CNT and Au degradation over long-term usage in future work to understand the effect on data reliability.

### CV data analysis

MATLAB was used to process CV data. Curves were first smoothed with a low-pass filter with a cutoff frequency of 3 Hz and a sampling frequency of 2 kHz (Supplementary Fig. S1). This was done mainly to allow accurate measurements of lower-concentration samples, which had a lower signal-to-noise ratio. Anodic peak current (Ipa) measurements were performed by calculating a linear regression fit for the background of each CV curve, subtracting this fit background from the full CV curve data and finding the maximum peak difference (Supplementary Fig. S2). This technique allows for more accurate measurement of small peaks, especially when blank CV controls (e.g., DMEM) do not perfectly overlap with the background of experimental CV curves. Repeats are shown as repeated CV cycles per one or two electrodes, and error bars were calculated as the standard deviation across repeats. Linear and nonlinear regression was performed to fit data to trendlines and curves.

### Cell culture

The RIN14B rat islet cell line (ATCC^®^ CRL2059™) was used as a surrogate for 5-HT-secreting enterochromaffin cells in the mammalian gut epithelium. Standard cell culture was performed in T75 polystyrene flasks from Thermo Fisher Scientific with incubation at 37 °C and 10% CO_2_. Cells were cultured in DMEM with 10% fetal bovine serum (FBS) (Thermo Fisher Scientific, Waltham, MA, USA). Cell passaging was performed every 3–5 days with 2.5% trypsin-EDTA (Sigma-Aldrich). RIN14B cells cultured in T75 flasks were used for 5-HT release experiments. Cells were first equilibrated by incubation with 5 mL DMEM + FBS and then stimulated to release 5-HT by treatment with 100 µM butyrate (Sigma-Aldrich) and incubation for 1 h. Cells were imaged with bright-field microscopy using an Olympus CKX53 inverted microscope.

RIN14B growth was attempted in our 3D-printed electrode-integrated in vitro platform, as described previously^46^. Cell attachment was encouraged by membrane treatment with type-I collagen (Sigma-Aldrich), which was coated according to the protocol. While RIN14B cells cultured in plastic flasks were able to be visually inspected by bright-field microscopy, the transwell devices were not optically transparent; thus, cell imaging was performed as an endpoint assay with live/dead fluorescent staining, including Syto9 and propidium iodide (PI) to stain live and dead cells, respectively. To achieve this, cells cultured on the membrane were first washed with Tris HCl buffer pH = 7.3, incubated with 3.4 mM Syto9 and 20 mM PI solution at room temperature for 15 min, and then washed again with Tris HCl buffer. Finally, pieces of the membrane were sectioned and sandwiched between a glass slide and a coverslip for visualization via reflection mode confocal microscopy. Excitation of both dyes was performed at 460 nm, and dual-mode emission detection was used to measure the 500- and 550-nm emission wavelengths. Syto9 and PI were obtained from the BacLight cell viability kit (Thermo Fisher Scientific). Confocal microscopy was performed with a Zeiss LS 700 laser-scanning confocal microscope. The autofluorescence of polystyrene prevented live/dead imaging of cells on T75 flasks, so morphology was compared between bright-field and fluorescence images (Fig. 8).

## Supplementary information

Supplementary Information
