# Supplementary material for: Electrochemical measurement of serotonin by Au-CNT electrodes fabricated on microporous cell culture membranes
Source: Microsyst Nanoeng. 2020 Sep 7;6:90. doi: 10.1038/s41378-020-00184-4 (PMC8433419; doi:10.1038/s41378-020-00184-4)
Supplement: Supplementary file 1 — Supplementary Information [file 41378_2020_184_MOESM1_ESM.docx]

# **ELECTROCHEMICAL MEASUREMENT OF SEROTONIN BY AU-CNT ELECTRODES FABRICATED ON MICROPOROUS CELL CULTURE MEMBRANES**

*A.A. Chapin^1^, P. Ramiah Rajasekaran^4^, D.N. Quan^1^, L. Hu^5^, J. Herberholz^6^, W.E. Bentley^1,2,3^, and R. Ghodssi^1,3,4,7*^*

^1^Fischell Department of Bioengineering, ^2^Institute for Bioscience and Biotechnology Research, ^3^Robert E. Fischell Institute for Biomedical Devices, ^4^Institute for Systems Research,

^5^Department of Materials Science and Engineering,

^6^Department of Psychology and Neuroscience and Cognitive Science Program,

^7^Department of Electrical and Computer Engineering,

University of Maryland, College Park, Maryland, USA

**Supplemental Information**

**Figure S1.** CV Data filtering with low pass filter: 3Hz cutoff frequency, 2kHz sampling frequency. a,b) 2mM FDM measured at Au-CNT microdisk electrode. a) Full CV. b) Zoomed CV. c,d) 1µM 5-HT measured at Au-CNT membrane electrode. c) Full CV. d) Zoomed CV.

**Figure S2.** Measuring anodic peak current (Ipa) by subtraction of fit curve (red, dashed) from CV data (blue, solid). a,b) 2mM FDM. a) Full CV. b) Zoomed CV, with diagram of background fitting and Ipa calculation. Green brackets denote data points surrounding the peak which were selected for linear regression of the background curve (power 3 polynomial fit), resulting in the red, dashed fit curve. The Ipa value is calculated by vertical subtraction of the background fit curve from the CV curve, and selecting the maximum difference of these two curves. c) DMEM cell media, no peak. d) 1µM 5-HT.


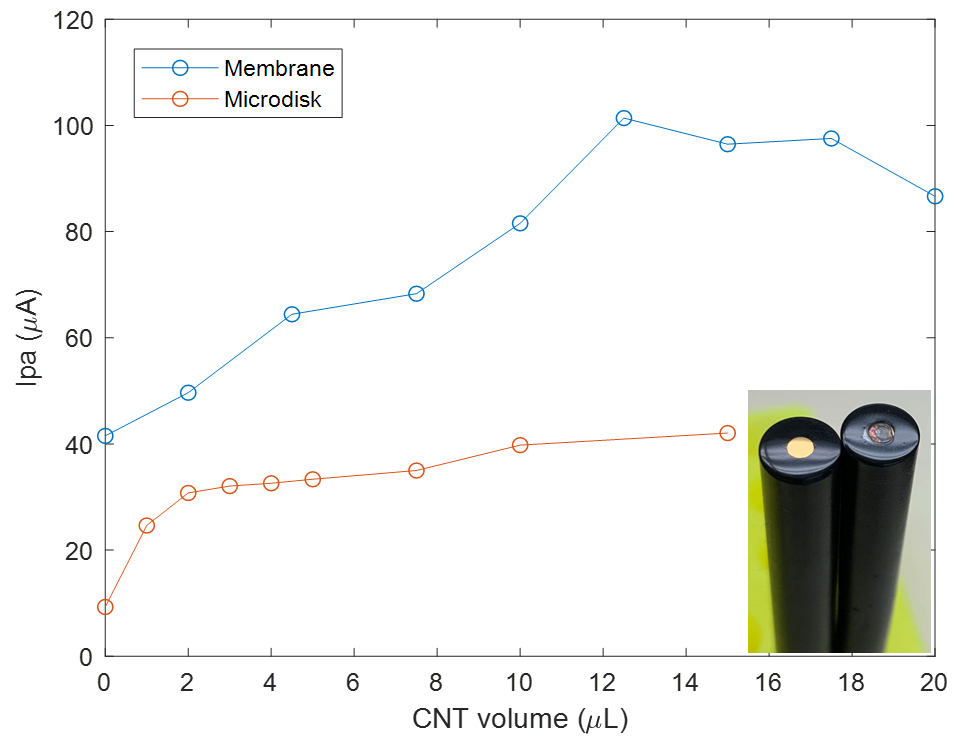


**Figure S3.** Optimization of CNT coating volumes on Au membrane and Au microdisk electrodes. CV Ipa peak height measured in 2mM FDM is reported for increasing volumes of CNT (Microdisk: 0 – 15µL, Membrane: 0 – 20µL).

**Evaluation of FigS3**

Microdisk electrodes are standard Au solid state electrodes, which serve as a good comparison to our thin film Au electrodes deposited on a porous, flexible PETE membrane. Supplementary figure S3 shows that increasing CNT volumes drop-casted on microdisk and membrane electrodes generally increases the anodic peak current response (Ipa) measured at the electrode in 2mM ferrocene dimethanol (FDM), which is used as a reference redox molecule. The Ipa signal plateaus at approximately 2µL of the CNT coating on microdisk electrodes, and approximately 12.5µL on membrane electrodes, owing to their different footprints (3mm^2^ and 7mm^2^ for microdisk and membrane electrodes, respectively). Thicker coatings, made with more than 12.5µL of the CNT solution, were less stable and more likely to flake off in solution.


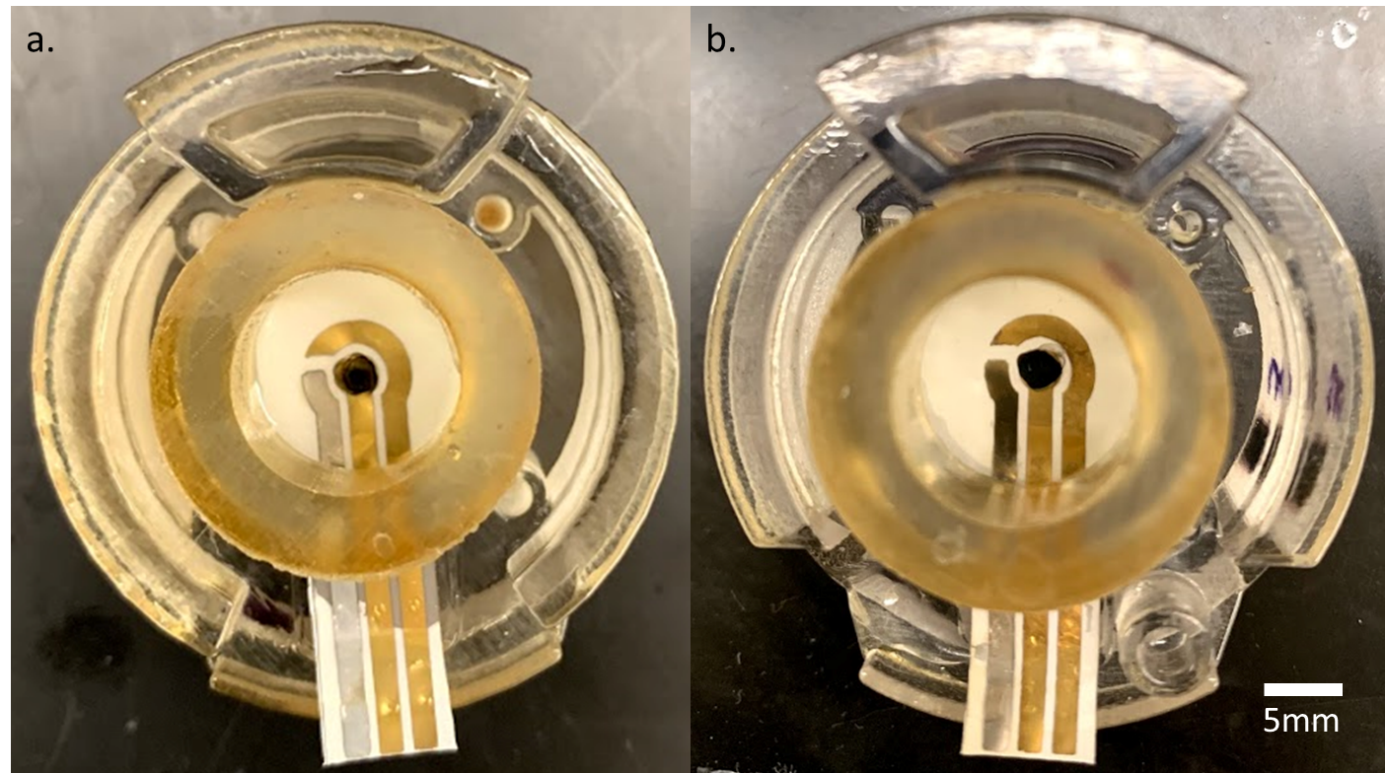


**Figure S4.** Images of the Au-CNT 2µL (a) and 12.5µL (b) membranes inside 3D printed housing.


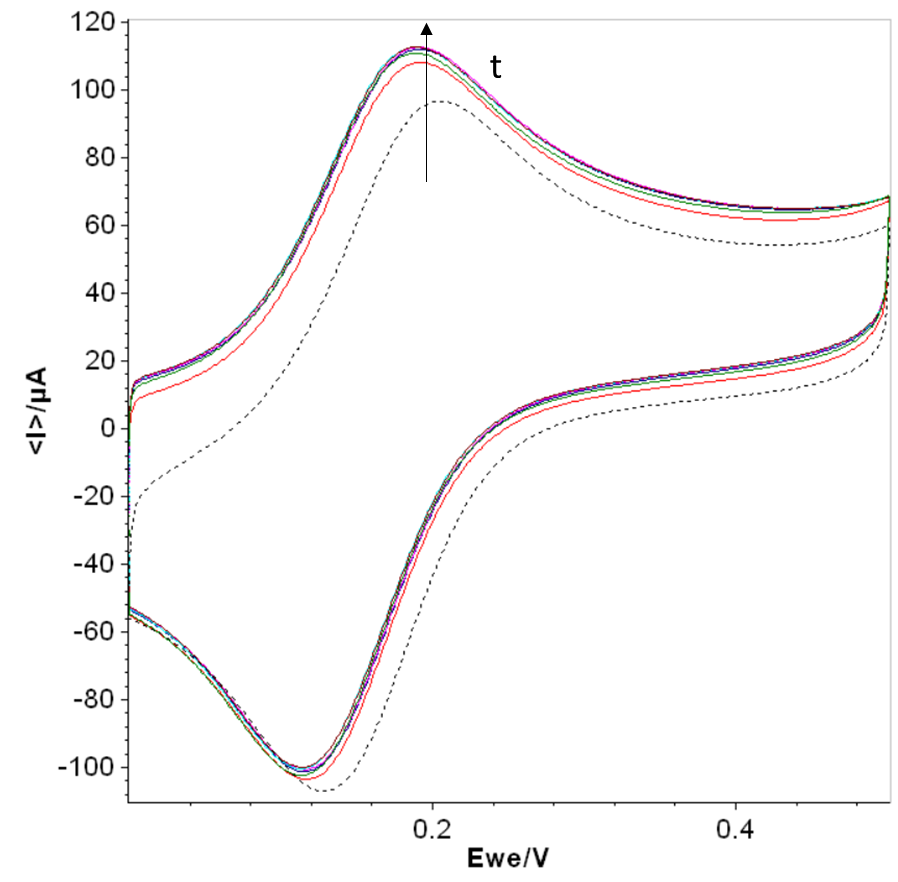


**Figure S5.** CVs of 2mM FDM at Au-CNT 12.5µL membrane electrode over a range of accumulation time: 0min - black dashed, 15s – 1.5min - colored lines. Signal saturates after 15s accumulation time.


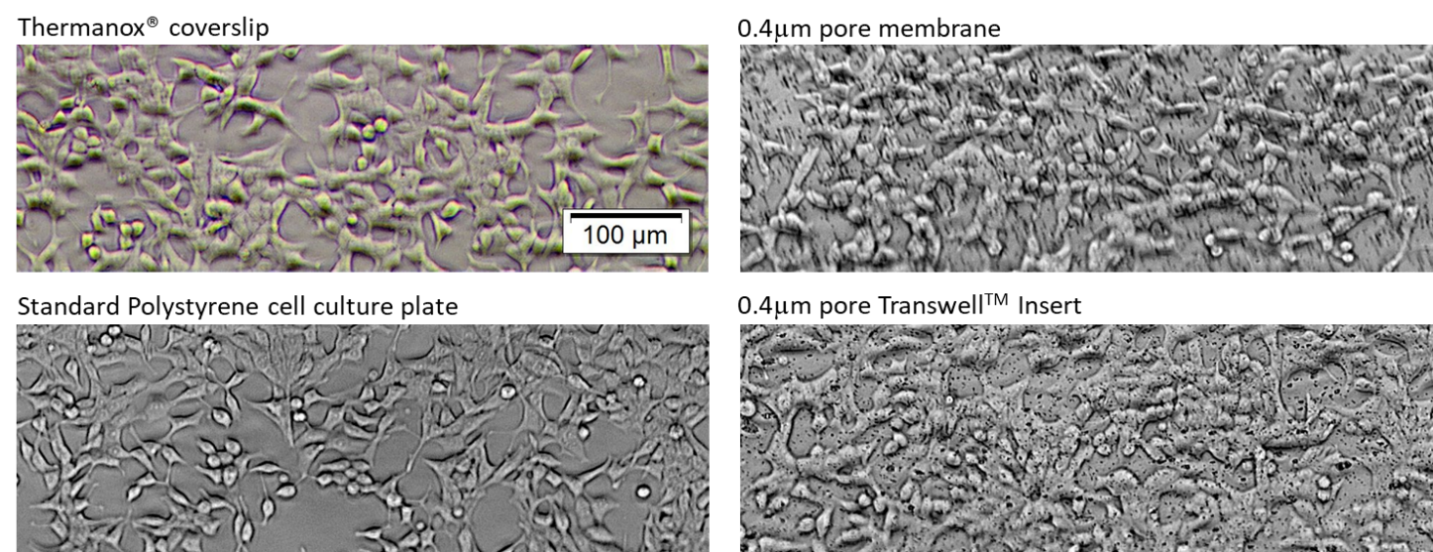


**Figure S6** Optical brightfield images of subconfluent RIN14B cultures on various transparent substrates. Scale bar applies to all images. NUNC^TM^ Thermanox^TM^ coverslips were obtained from Thermo Fisher.

**Cell Mechanobiology of Attachment**

LIVE/DEAD staining of RIN14B monolayers grown on the 1µm pore transwell membrane used in this study (Fig. 8) gives evidence that cells are rounded, which is a sign of decreased attachment of cells to their substrate. Supplementary Figure S6 shows that RIN14B cells seem to attach much better to both polystyrene and PETE with low porosity and 400nm pore sizes. Both of these substrates provide more surface area for attachment than the higher porosity PETE membranes with 1µm pore sizes. Cells cultured on this porous membrane were observed to easily detach, even during gentle washes, further indicating that cells are not strongly attached. Cell attachment is mediated by integrin binding to ECM proteins either existing on the substrate, or secreted by the cells themselves. It is this binding that allows the cell to spread over its surface and form focal adhesions, which modulate the cytoskeleton to both form stable mechanical adhesion and regulate intracellular signaling.^1^ Integrins and other protein complexes are shown to control the polarization of epithelial cells, which is essential for proper orientation of the microtubule network and vesicle transport.^2^ Therefore, poor cell attachment could be considered a cause of inhibited secretory granule release, the process needed for 5-HT secretion.

**Instrumentation Approaches**

The 1µm pore PETE membrane was chosen for its high porosity, which allows rapid molecular transport across the membrane. While 400nm pore PETE membranes with low porosity show significantly enhanced cell adhesion, the molecular transport is much slower, which would not be beneficial for dynamic molecular detection. Other material and fabrication methods can be explored to customize an optimal level of porosity to balance cell adhesion and molecular transport. For instance, two photon polymerization (TPP) 3D printing can deposit biocompatible materials with microscale feature sizes, allowing rapid prototyping with varying porosity.^3^ These surfaces can also be plasma activated to create more acidic groups for electrostatic cell interaction,^4^ or coated with different ECM proteins such as those used to connect epithelial cells to the basal lamina (e.g. laminin, collagen IV).^5^

**References**

1. Khalili, A. A. & Ahmad, M. R. A Review of Cell Adhesion Studies for Biomedical and Biological Applications. *Int. J. Mol. Sci.* **16**, 18149–18184 (2015).

2. Lee, J. L. & Streuli, C. H. Integrins and epithelial cell polarity. *J. Cell Sci.* **127**, 3217–25 (2014).

3. Lee, J.-Y. *et al.* The potential to enhance membrane module design with 3D printing technology. *J. Memb. Sci.* **499**, 480–490 (2016).

4. Wang, D.-Y., Huang, Y.-C., Chiang, H., Wo, A. M. & Huang, Y.-Y. Microcontact printing of laminin on oxygen plasma activated substrates for the alignment and growth of Schwann cells. *J. Biomed. Mater. Res. Part B Appl. Biomater.* **80B**, 447–453 (2007).

5. Arends, F. & Lieleg, O. Biophysical Properties of the Basal Lamina: A Highly Selective Extracellular Matrix. in *Composition and Function of the Extracellular Matrix in the Human Body* (InTech, 2016). doi:10.5772/62519
